# A multi-biomarker approach to assess the exposure effects of MPs and/or sodium lauryl sulfate on *Clarias Gariepinus*

**DOI:** 10.1038/s41598-025-21773-1

**Published:** 2025-12-08

**Authors:** Alyaa Elsayed, Hamdy A. M. Soliman, Fatma Ahmed, Hanem S. Abdel-Tawab, Alaa El-Din H. Sayed

**Affiliations:** 1 Department of Zoology, Faculty of Science, Sohag University, Sohag, 82524 Egypt; 2 Department of Zoology, Faculty of Science, Assiut University, Assiut, 71516 Egypt; 3 Molecular Biology Research and Studies Institute, Assiut University, 71516 Assiut, Egypt

**Keywords:** PMPs, Superoxide dismutase, SLS, Liver, Spleen, fibrosis, Hypoglycemia, Zoology, Biomarkers

## Abstract

Pollutants impact fish health, leading to environmental diseases. Anionic surfactant detergents and MPs (MPs) consist serious threat in freshwater environments either lonely or in combination. However, their combined effects are not well studied. Our research therefore focuses on studying the harmful effects of the anionic surfactant sodium lauryl sulfate (SLS) and polyethylene MPs (PMPs) on the biology of freshwater African catfish (Clarias gariepinus), either alone or in combination. A 15-day exposure trial to PMPs (10 mg/L), SLS (4 mg/L), or their combination was conducted. Hematological, biochemical, antioxidant, and immunological markers were estimated. The erythrocytic morphology was investigated. The pathological harms were monitored, and the histological abnormalities were scored. In addition, histochemical appraisals of fibrosis and hypoglycemia in the liver and spleen were estimated. This was achieved by semi-quantification of polysaccharide deposits and the fibrotic collagen density and distribution pattern in the tissue micro-sections. On the one hand, our findings revealed deteriorated biological markers in C. gariepinus exposed to PMPs (10 mg/L) or SLS (4 mg/L) by close levels. Significant decreases in the hematological indices, while substantial increases in the biochemical markers were recorded. As well, significant decreases were recorded in the total antioxidant capacity and superoxide dismutase, while an elevation was recorded in the level of IL-1β and IL-6 cytokines. Poikilocytosis of erythrocytes and severe hepatic and splenic pathological lesions were observed. Furthermore, high levels of fibrosis and hypoglycemia were detected. On the other hand, our findings showed antagonistic effects upon the combined exposure to PMPs (10 mg/L) and SLS (4 mg/L). Fluctuated non-significant differences were observed in hematology and biochemical markers. Mild erythrocytic poikilocytosis and moderate pathological lesions were scored in liver as well as spleen. In addition, moderate quantitative fibrosis, and hypoglycemia were estimated. Exposure to PMPs and SLS deteriorate the biology and pathology of C. gariepinus by severe effects. Interestingly, ameliorated biological alterations were recorded evidenced a surprising antagonistic effect of PMPs + SLS. Possibly, a chemical chelation between both reagents counteracted their singular effect inside the biological system, which retorted their harm. Therefore, further investigations by chemists on the probable chemical interaction between PMPs and SLS inside biological systems, which might change their physical or chemical characteristics, may explain the case.

## Introduction

 Aquatic pollutants produced by anthropogenic activities are the primary cause of fish’s disorders. They alter the biology, physiology, and immune defense status of exposed fish, which in turn deteriorates their resistance to infections^1,2^. Additionally, many environmental pollutants are toxicants and act, at once, as hidden carriers for fish pathogens transferring them deeply inside the living tissues^3,4^. Physiologically, pollutants in the aquatic environment can alter fish hematological, biochemical, and molecular markers. Therefore, hematological and biochemical indices were employed as bioindicators of abnormalities since the blood is the primary pathway via which a material enters the fish^5,6^. In addition, sensitive indicators of structural changes within cells and tissues of some organs are provided by the histopathological and histochemical testing techniques^7^.

Plastics are widely utilized in manufacturing to improve strength, lower product weight, and provide transparency for applications such as displays^8^. Polymerizing monomers like ethylene and propylene create plastics known as synthetic and semisynthetic materials^9^.In greater demand and several uses, they have raised humankind’s standard of living^10^. Non-biodegradable plastics are widely used, have contaminated the environment, and progressively accumulated and transmitted through the food chains from animals to humans. Microplastics (MPs) are recently described pollutants that have gained international notice and are now a topic of study in aquatic environmental science. While MPs come from several sources, primary and secondary sources are the frequent pathways they reach the environment that are strongly linked to the widespread use of MPs and their existence^11,12^. The primary sources are the microbeads of micro-sized plastic particles added to personal care products like toothpaste, face and hand cleaners, etc^11,13^. The secondary sources are those micro-sized plastic particles resulting from the physical, chemical, or biological fragmentation of macro- and meso-sized plastic products^14^. The enormous input of plastics is considered the primary reason for the contamination of aquatic farms with MPs^15^. These MPs are abundantly accumulated in the farmed aquatic species, including fish, which construct the most suppliers of high-quality protein in Egypt’s aquaculture that produce > 50% of commercial supply^16–18^. The bioaccumulation of MPs is more easily due to exogenous factors, such as plastic fishing tools, industrial farming facilities, and equipment, or endogenous factors, such as animal health fortifiers, and feed additives.

Owing to their bioaccumulation, several studies have reported that MPs can elicit eco-toxicological effects in fish, such as anemia, biochemical disturbance, and oxidative stress^19^. The histopathological hazards of MPs on fish tissues have been extensively documented and primarily cause inflammations and oxidative stress^20–23^. Furthermore, fish residues from MPs can pose a variety of risks on aquaculture safety and intimately linked to human health as it has been discovered in human feces^24,25^. Moreover, because MPs have a strong affinity for hydrophobic chemicals persist in water, they can not only directly harm aquatic organisms, but they can also act as concentrators and transporters of harmful substances^22,26^.

Surfactants are classified as hazardous toxicants among all other types of pollutants based on their hydrophilicity, which produces anionic, cationic, or non-ionic substances^27^. They are extensively utilized in a wide range of products, including detergents, personal care products, and household cleaners, paints and dyes, textiles, herbicides, medicines, oils, foods, cosmetics, papers, rubbers, and metal processing^28^. Usually, surfactants persist in aquatic ecosystems in relatively low quantities, ranging from a few µg L^−1^ to mg L^−1^^29^. One common anionic surfactant is sodium lauryl sulfate (SLS) or sodium dodecyl sulfate (SDS), which is a primary alkyl sulfate belonging to the alcohol phosphate family^30^. It is highly biodegradable and has a low bioaccumulation rate and does not linger in the environment for long period, therefore it is formally categorized as “environmentally friendly”^31^. Nonetheless, some research has indicated that SLS may be fatal in short-term exposures^32^. It has been shown that SLS is toxic to fish, echinoderms, bacteria, and microalgae^33^.

Noteworthy, the different chemical substrates in the same ecosystem might interact antagonistic or synergistic^34^. Interactions between MPs and ambient chemical contaminants were reported in natural water systems^35^. Therefore, combined effects of chemically hazardous pollutants, such as metal ions, detergents, agrochemical pesticides, and MPs on a variety of aquatic animals, mostly fish, have been the subject of various studies conducted more recently^36,37^.

Recently, our group conducted deep investigations on the nephrotoxicity of PMPs and/or SLS on the African catfish (*Clarias gariepinus*)^38^. *C. gariepinus* is a common freshwater fish species mostly desirable for aquaculture, and it is a suitable fish model frequently utilized in the biological investigations of toxicological research^39^. Therefore, the current study employed *C. gariepinus* as a fish model to evaluate the biological and physiological alterations caused by singular and/or combined exposure to nonlethal doses of PMPs and SLS. Several biological indicators of hematological, biochemical, and antioxidant indices were estimated. In addition, some histopathological and histochemical biomarkers were investigated. Here, semi-quantification of pathological traits, fibrosis, and hypoglycemia in fish liver and spleen highlighted the antagonism between MPs and SLS inside the biological tissues. This opened new horizons for further in-deep investigations of the possible chemical chelation between MPs and SLS under water ecosystems in general and inside the biological systems specifically. This antagonistic interaction between MPs and SLS summarizes the novelty of the current investigation.

## Materials and methods

### Chemicals

PMPs raw powder was purchased from the Toxemerge Pty Ltd. Company (Melbourne, Australia). SLS (> 99% purity) was purchased from Sigma–Aldrich Chemical Co. (St. Louis, MO, USA). Kits for the analysis were bought from Bio-Diagnostic Co., Cairo, Egypt.

### Stock preparation and characterization of MPs (MPs)

The manufacturer’s protocol was followed in preparing the stock solution (1 g PMPs/L), which was then kept in the dark at 4 °C, and sonicated using Milli-Q purified water before use. Just before starting each experiment, this stock was further diluted for test concentrations. Scanning electron microscopy (SEM, JEOL JEM-1200 EX II) was used to characterize the morphology of PMPs at Assiut University.

### Experimental design

#### Ethics statement

The in vivo experimental protocol (CSRE-37-24) and the fish handling procedures were approved by the Research and Ethical Committee of the Faculty of Science, Sohag University, Sohag, Egypt. All methods were carried out in accordance with relevant guidelines and regulations. All methods are reported in accordance with ARRIVE guidelines.

#### Fish exposure

Purchased from an authorized private farm, healthy *C. gariepinus* with an average body weight of 250–300 g and an average length of 25–30 cm was delivered to the wet Laboratory of Fish Biology and Pollution, Faculty of Science, Assiut University. The fish were checked for pathogen-free status to ensure their healthy condition^40^.

Before starting the experiment, the fish were examined to ensure they were healthy and clear of pathogens. They also spent four weeks becoming used to the lab environment. Fish were reared in 100-liter fiberglass tanks with pH = 7.4, dissolved oxygen = 6.9 mg/L, and a temperature of 20 °C in dechlorinated tap water. After 2 weeks of acclimation to a 12:12 h light/dark photoperiod, fish were given a commercial basal diet at 3% body weight twice daily.

For a 15-day toxicant exposure testing, 64 *C. gariepinus* were divided into four groups in duplicate (8 fish/tank; 16 fish/group). Fish that had not been exposed to toxins were in the first group, called Control, while the fish that had been exposed to toxicants comprised the other three categories. The second group, known as PMPs-exposed, was exposed to 10 mg/L of PMPs; the third group, known as SLS-exposed, was subjected to 4 mg/L of SLS; and the fourth group, known as PMPs + SLS-exposed, was exposed to 10 mg/L of PMPs + 4 mg/L of SLS. The hazardous doses reported in earlier research guides our selection of the exposure dosages of the current study. Besides, our selected doses ensure fish safety throughout the 15-day acute exposure period^41,42^. As directed by their manufacturer, stock solutions comprising 1 g/L of PMPs or SLS in ultrapure water (Milli-Q) were designated and maintained in the dark at 4 °C for our investigations. To adjust the measured exposure doses, the stock solutions were quickly diluted in the new rearing water and mixed before each rearing water exchange^43^. After two days of the prior exposure, the exposure regimen was repeated by replacing half of the water in the tanks and doing the PMPs and/or SLS again. All groups were maintained in the same acclimation setup and fed according to the same regimen (3% body weight) for 15 days of exposure.

#### Blood biological and immunological indices

I. *Hematological Parameters*.

The following hematological parameters were measured following^44^, mean corpuscular volume (MCV), mean corpuscular hemoglobin (MCH), mean corpuscular hemoglobin concentration (MCHC), hemoglobin (Hb), Hematocrit (HCT), red blood cell count (RBCs), and the differential count of white blood cells (WBC).

II. *Biochemical Parameters*.

Using kits from SG Mitalia Company (U.S.A.) and a spectrophotometer (T80 + UV/VIS, Bioanalytic Diagnostic Industry, Co.), total protein, glucose, cholesterol, aspartate aminotransferase (AST), alanine aminotransferase (ALT), and alkaline phosphatase (ALP) were measured in fish serum^43,45^. *Antioxidant Parameters*.

Serum samples were used to evaluate the total antioxidant capacity (TAC) and SOD activities using previously published techniques^46–48^. The thiobarbituric acid reaction was used to calculate the malondialdehyde (MDA) level^49^.

III. *Inflammatory Signals (Cytokines)*.

According to Soliman et al.^50^, blood cytokines (interleukin-6 “IL-6” and interleukin-1β “IL-1β”) were quantified in the fish sera commercially using a highly sensitive ELISA set (Human Ultrasensitive, BioSource International Inc.).

IV. *Erythrocyte Morphology*.

Blood smears of all groups were prepared, fixed, and air-dried. Erythrocytic differentiation was achieved using Hematoxylin & Eosin (H&E) staining. Visual observations of cell morphology under a light microscope (VE-T2) were documented using an attached camera (14 MP OMAX) (A35140U3)^43,45^.

#### Histopathology and histochemistry

Small pieces of liver and spleen were meticulously gathered, cleaned, and fixed in 10% neutral buffered formalin for 48 h. They were then dehydrated in ascending concentrations of ethanol, cleared in xylene, wax embedded, and sectioned at 5 μm. Dewaxing in xylene, and staining for the microscopic examination using Harris’s H&E counter stain was conducted to differentiate tissue degenerations and/or cell death^51^. The semi-quantitative pathological comparison was conducted as described by^52^ with minor modification. Triplicate tissue sections of each group were examined under an Olympus microscope (BX50F4, Olympus Optical Co., LTP, Japan) and the collective structural damages and abnormalities observed under 40x magnification power in three fields per section (*n* = 9) were monitored and graded as 0 for the normal structure or 1–3 for the gradual severity appraisals (mild, moderate, or severe). The obtained data were manipulated statistically and represented in bar histograms as means ± SE. In addition, a differential lesion severity grading was assessed for each organ according to Fernandes protocols with some modifications^53^. The differential severity levels represent the percent of existing trait of a lesion in the nine fields examined per organ. The individual lesion severity was graded in 3 levels according to its frequency. The obtained data were expressed as mild (+), moderate (++), or severe (+++).

Special staining with Sirius Red (SR) stain was used to detect fibrosis^54^, and with Periodic acid Schiff reaction (PAS) was used for the detection of carbohydrate content, primarily glycogen, was carried out following the routine protocol^55^. With slight modifications to Saleh et al.‘s semi-quantitative assessments^56^, collagen type I and III fibers in SR-stained sections of the liver, and spleen were statistically quantified to estimate tissue fibrosis using ImageJ software (version 1.41o, Public Domain, BSD-2, https://imagej.net/Ops). The software processes were applied to eighteen (*n* = 18) triplicate randomly selected digital images (taken at 40×) of the sections from each dissected fish. For every group, two characteristics were displayed in semi-quantification: (i) the growing pattern of these fibers in the whole area (%) and (ii) the density of fibers per 0.5 mm^2^ area of each image that was chosen. The acquired information was represented as Means ± standard error and shown in histograms. Similarly, the total positively stained area (%) of collagen deposits in randomly chosen digital pictures of PAS-stained sections of the liver from each group (acquired at 40×, *n* = 18) was semi-quantified.

### Statistical analysis

The obtained data were checked using Pearson’s chi-squared test for normality, and the values clustered around the mean, indicating a homogeneous distribution suitable for statistical comparison. Simple descriptive statistics were used to obtain the means and standard error (SE). Duncan’s multiple range test (MRT) and a one-way analysis of variance (ANOVA) were used to assess the significance of the mean and standard error comparison. All statistical analyses were conducted using the statistical package for social sciences (SPSS) software (version 17.0 for Windows 10), with a significance threshold of 0.05. P-values of less than 0.01 (**) are regarded as highly significant, and less than 0.001 (***) as very highly statistically significant^57^.

## Results

### Surface characteristics of PMPs

**Fig. 1:**
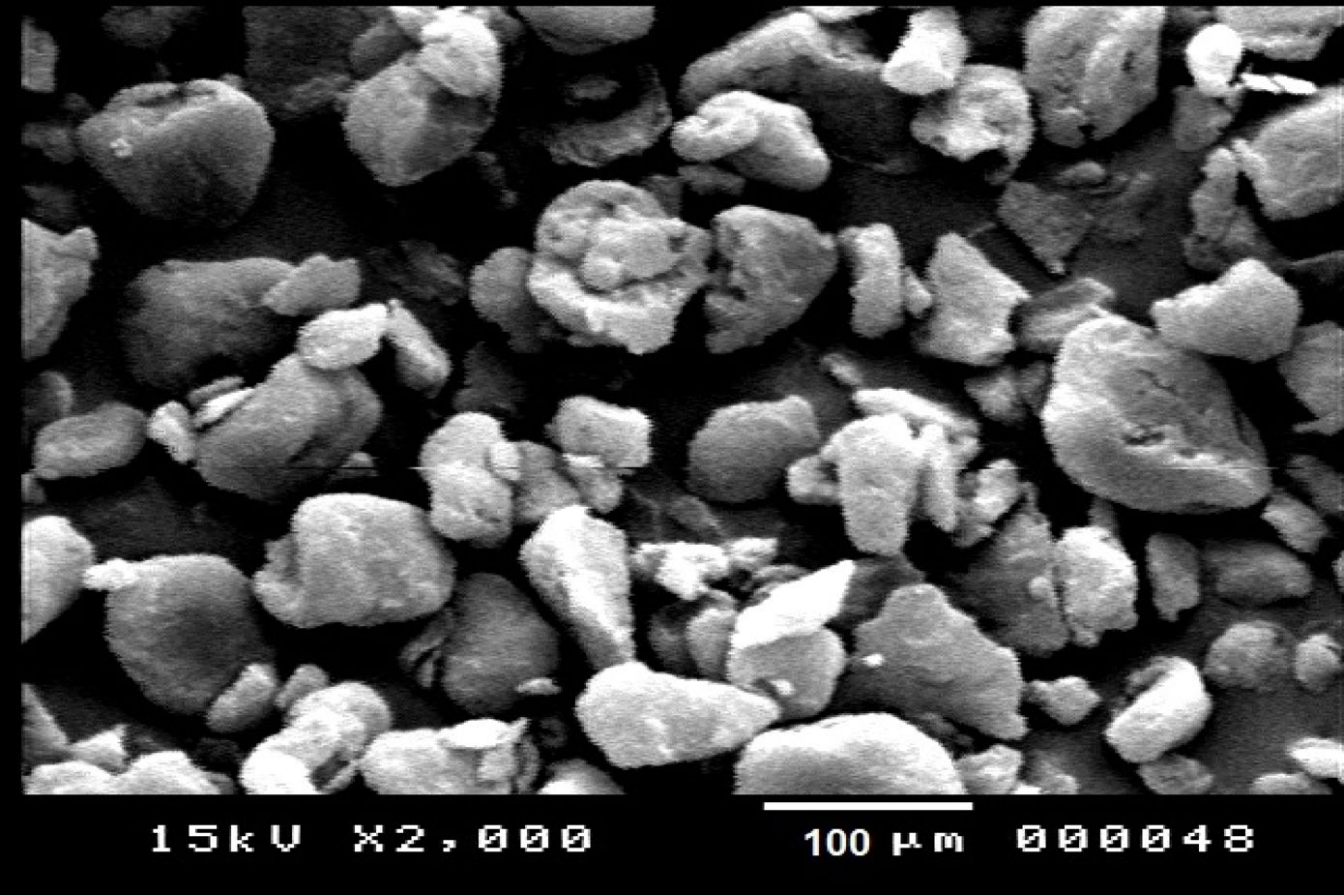
Photomicrograph displays the irregular-shaped particles of the PMPs in water medium under a scanning electron microscope (JEOL JEM-1200 EX II).

The PMPs have uneven shape and size as shown by the SEM micrography in Fig. 1.

### Hematological indices

#### Hematological parameters

The hematological indices of RBCs, blood hemoglobin concentration (Hb), hematocrit (Ht), Platelets, and total (WBC), showed a significant (*P* < 0.05) decrease after exposure to PMPs, or SLS, or their combination for 15 days in comparison with the control group. Reversely, MCV level showed a significant increase (*P* < 0.05) in the SLS-exposed group while the levels of MCHC and MCH showed no significant change after exposure to PMPs, or SLS, or their combination for 15 days.

The differential count of WBC showed a significant decrease (*P* < 0.05) in lymphocyte and monocyte percent, while a significant (*P* < 0.05) increase in eosinophil and neutrophil percent after exposure to PMPs, or SLS, or their combination for 15 days compared to the control group (Table 1).

**Table 1:** Single and combined effect of 14-days exposure to 10 mg/L polyethylene MPs (PMPs), and 4 mg/L sodium lauryl sulfate (SLS) on the hematological parameters of the African catfish (*Clarias gariepinus*).

Parameter	Control	PMPs	SLS	PMPs + SLS
RBC’s (Million/mm^3^)	3.04 ± 0.06^a^	2.61 ± 0.04^b^	2.60 ± 0.04^b^	2.54 ± 0.04^b^
Hb (g/dL)	8.81 ± 0.4^a^	7.3 ± 0.3^b^	7.6 ± 0.2^b^	7.1 ± 0.3^b^
Ht (%)	33.96 ± 0.2^a^	30.91 ± 0.21^b^	30.91 ± 0.2^b^	30.19 ± 0.2^c^
MCV (µm³)	106 ± 1.4^a^	113 ± 1.7^b^	113 ± 1.7^b^	111 ± 1.7^b^
MCH (Pg)	27.4 ± 0.7^a^	26.6 ± 1.0^a^	26.6 ± 1.02^a^	26.03 ± 0.9^a^
MCHC (%)	24.6 ± 0.9^a^	22.4 ± 0.95^a^	22.4 ± 0.5^a^	21.9 ± 0.9^a^
Thrombocytes (Thousands/mm^3^)	211 ± 3.9^a^	198 ± 1.7^b^	198 ± 1.7^b^	193 ± 1.6^b^
WBC’s (Thousands/mm^3^)	11.2 ± 0.2^a^	10.4 ± 0.1^b^	10.4 ± 0.1^b^	10.1 ± 0.1^b^
Neutrophils (%)	11.5 ± 0.2^a^	13.5 ± 0.2^b^	13.3 ± 0.3^b^	13.8 ± 0.2^b^
Large lymphocyte (%)	58.8 ± 0.2^a^	55 ± 0.4^b^	54 ± 0.5^bc^	53.8 ± 0.4^c^
Small lymphocyte (%)	25.2 ± 0.2^a^	21.8 ± 0.3^b^	22.3 ± 0.3^b^	22.3 ± 0.3^b^
Monocyte (%)	3.7 ± 0.2^a^	2.5 ± 0.2^b^	2.7 ± 0.3^b^	3.3 ± 0.2^a^
Eosinophils (%)	2.8 ± 0.2^a^	7.1 ± 0.4^b^	7.3 ± 0.2^b^	6.8 ± 0.2^b^

* Data are represented as means ± SE (n = 3). Values with different superscript letters in the same row for each parameter are significantly different (*P* < 0.05). Hb: Hemoglobin, Ht: Hematocrit, MCV: Mean corpuscular volume, MCH: Mean corpuscular hemoglobin, and MCHC: Percentage of mean corpuscular hemoglobin concentration.

#### Biochemical parameters

The liver functions showed no significant change in aspartate aminotransferase (AST) level, and a significant (*P* < 0.05) increase in alanine aminotransferase (ALT) level, while a significant (*P* < 0.05) decrease in alkaline phosphatase (ALP) level. The other biochemical parameters of glucose, cholesterol, and total protein levels showed a significant (*P* < 0.05) increase after exposure to PMPs, or SLS, or their combination for 15 days in comparison with the control group (Table 2).

**Table 2:** Single and combined effect of 14-days exposure to 10 mg/L polyethylene MPs (PMPs), and 4 mg/L sodium lauryl sulfate (SLS) on the biochemical parameters of African catfish (*Clarias gariepinus*) blood.

Parameter	Control	PMPs	SLS	PMPs + SLS
AST (U/L)	32.4 ± 0.8^a^	33.4 ± 0.6^a^	33.4 ± 0.6^a^	32.6 ± 0.6^a^
ALT (U/L)	16.01 ± 0.4^a^	17.2 ± 0.3^b^	17.2 ± 0.3^b^	16.8 ± 0.3^ab^
ALP (U/L)	44.5 ± 1.5^a^	40.7 ± 0.99^b^	40.7 ± 0.99^b^	39.8 ± 1.0^b^
Glucose (mg/dL)	69.2 ± 1.0^a^	74.99 ± 0.4^b^	74.99 ± 0.41^b^	73.3 ± 0.5^b^
Total protein (TP) (mg/dL)	3.89 ± 0.1^a^	4.5 ± 0.2^b^	4.5 ± 0.2^b^	4.4 ± 0.2^ab^
Total Cholesterol (mg/dL)	201 ± 0.9^a^	215 ± 2.4^b^	215 ± 2.4^b^	210 ± 2.3^b^

* Data are represented as means ± SE (n = 3). Values in the same row with different superscripts are significantly different (*P* < 0.05). AST: Aspartate aminotransferase, ALT: Alanine aminotransferase, and ALP: Alkaline phosphatase.

### Oxidative stress biomarkers

The data presented in Table (3) showed the results of the investigated serum oxidant and antioxidant status of *C. gariepinus.* The total antioxidant capacity and SOD showed significant (*P* < 0.05) decreases in the SLS-exposed group while the Malondialdhyde level showed significant increases (*P* < 0.05) after the exposure to PMPs, or SLS, or their combination for 15 days compared to the control group.

**Table 3:** Single and combined effect of 14-days exposure to 10 mg/L polyethylene MPs (PMPs), and 4 mg/L sodium lauryl sulfate (SLS) on the antioxidant enzymes and lipid peroxidation in African catfish (*Clarias gariepinus*) blood.

Parameter	Control	PMPs	SLS	PMPs + SLS
Superoxide dismutase (SOD) (U/mL)	2.6 ± 0.1^a^	1.9 ± 0.1^b^	1.9 ± 0.1^b^	1.8 ± 0.1^b^
Total antioxidant capacity (nmol/L)	52.1 ± 3.1^a^	41.2 ± 0.89^b^	41.2 ± 0.89^b^	40.2 ± 0.86^b^
Malondialdehyde (nmol/mL)	15 ± 1.0^a^	28.6 ± 2.2^b^	28.6 ± 2.110^b^	27.9 ± 2.1^b^

* Data are represented as means ± SE (n = 3). Values with different superscripts in the same row are significantly different (*P* < 0.05).

#### Erythron profile

**Fig. 2:**
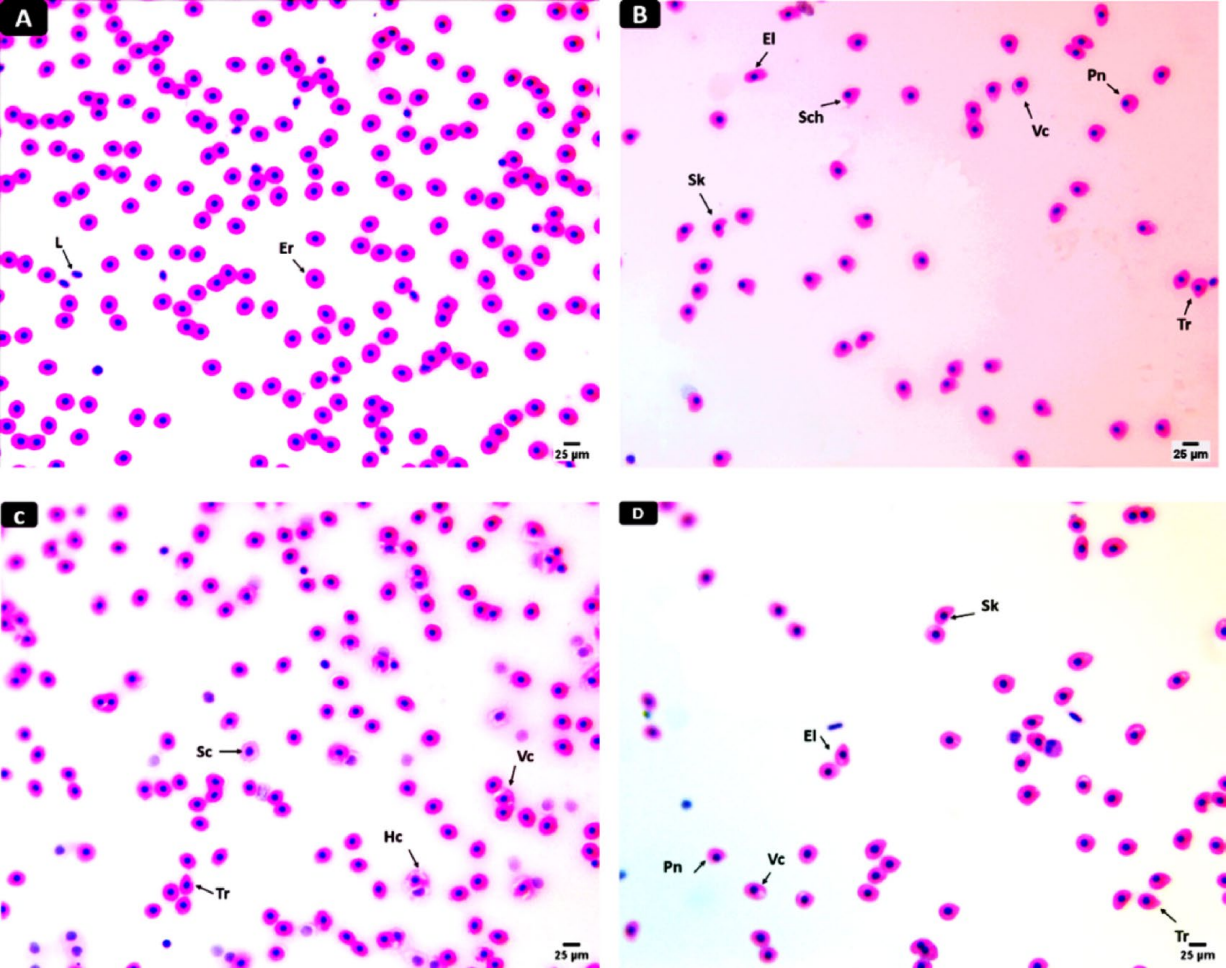
Micrographs of blood smears stained with H & E (×400; Scale bar = 25 μm) displaying normal and deformed erythrocytes following exposing C. gariepinus to PMPs, SLS or their combination for 15 days. A) Normal erythrocytes, B – D) deformed erythrocytes containing (Er) erythrocytes, (L) leucocytes, (Tr) teardrop cell, (Pn) peripheral nucleus, (Hc) hemolyzed cell, (El), elliptocyte, (Shc) schistocyte, (Vc) vacuolated cells, (Sc) swollen cells, and (Sk) sickle cell.

Blood smears from the control fish revealed normal erythrocytic morphology with a central nucleus and leucocytes (Fig. 2 , A). Alterations of erythrocyte shape (poikilocytosis) were observed in the blood smears of fish exposed to PMPs (10 mg/L) in the form of schistocytes, sickle cells, and elliptocytes, tear drop–like cells, vacuolated cells, peripheral nucleus. Smears from fish exposed to SLS (4 mg/L) showed poikilocytosis of erythrocytes including swollen cells, hemolyzed cells, –teardrop cells, and vacuolated cells. The blood from fish exposed to combined PMPs + SLS-exposed group showed erythrocyte poikilocytosis including elliptocytes, tear drop–like cells, vacuolated cells, peripheral nucleus, and sickle cells (Figs. 2 B–D).

### Histological analyses

#### Tissue integrity

I. Hepatic Pathology.

**Fig. 3:**
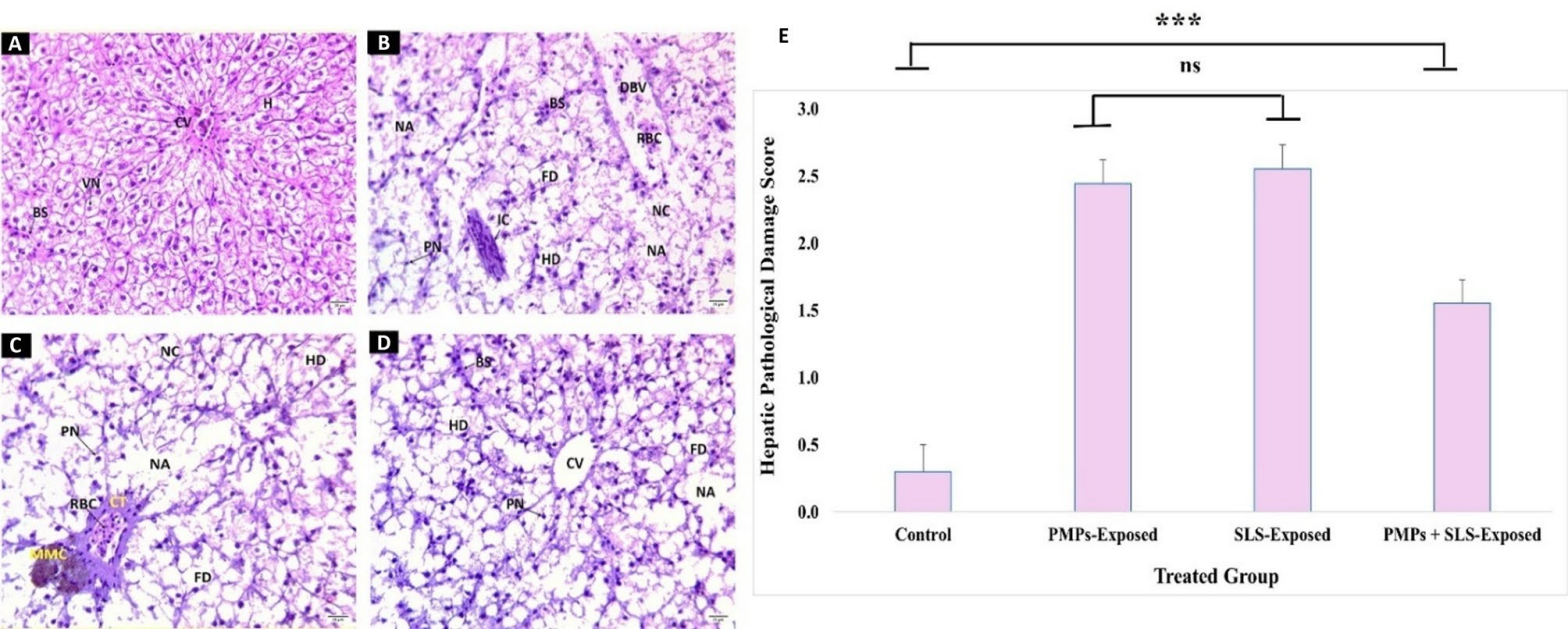
Photomicrographs of H & E-counterstained sections (×400; scale bar = 25 μm) of *C. gariepinus* liver after 15 days of exposure to PMPs, SLS, or their combination. **(A)** A micrograph from a control fish demonstrating typical hepatic integrity of H, VN, CV, and BS. **(B)** A micrograph from a fish subjected to PMPs demonstrates complete HD of hepatic cells with the appearance of IC, RBC, PN, NC, FD, congested BS, NA, and DBV. **(C)** A micrograph from a fish exposed to SLS displays severe deterioration in hepatic tissue appearing as HD, NA, RBC, PN, and FD. Wide MMC were obvious in CT surrounding the HVs rich in RBC. **(D)** A micrograph from a fish subjected to PMPs + SLS shows modest histological assessments of the hepatic tissue. HD, NA, PN, FD, and congested BS were noticed around the CV. **(E)** A bar graph displaying the severity score of the general hepatic histopathological lesion ranging from 0 to 3. The data were estimated at 40x magnification and displayed as means ± SE of nine replicates for each group (*n* = 9). The statistically significant differences in values between groups (*P* < 0.05) are indicated by superscript symbols on the bars.

As shown in Fig. 3, A liver the control group showed polygonal hepatocytes (H) containing fine-granulated acidophilic cytoplasm with vesicular nuclei (VN), central vein (CV), blood sinusoids (BS), and hepatic veins (HVs). Liver sections from PMPs-exposed fish (Fig. 3, B) showed hydropic degeneration (HD) of whole H, enormous necrotic area (NA), many degenerated or necrotic cells (NC), many pyknotic nuclei (PN) fatty deposition (FD), foci of inflammatory cells (IC), increased BS, and dilated blood vessels (DBV). Liver sections from SLS-exposed fish (Fig. 3, C) showed severe degeneration in hepatic structure, degenerated or necrotic hepatocytes (NC), increased NA, HD, PN, unclear BS, and melanomacrophage centers (MMC) beside congested central vein (CCV). In addition, full red blood corpuscles (RBC) which are surrounded by thick connective tissues (CT) and few FD were also observed. Liver sections from fish exposed to combined doses of MP (10 mg/L) + SLS (4 mg/L) (Fig. 3, D) showed HD, NA, NC, degenerated hepatocytes (DH), PN, FD, and increased BS. The scoring of the general pathological lesions observed in fish liver is represented in a column histogram (Fig. 3, E). In addition, the differential scoring of the observed pathological lesions is collected in Table 4.

**Table 4:** The differential severity scoring of the observed hepato-pathological lesions after the exposure to PMPs and/or SLS.

Lesion	Group/Score			
	Control	PMPs-Treated	SLS-Treated	PMPs + SLS-Treated
Hepatic cell degeneration (HD)	-	+++	+++	+++
Enriched inflammatory cells (IC)	-	+++	++	+
Enriched red blood corpuscles (RBC)	+	+++	+++	+
Enriched pyknoses nuclei (PN)	-	+++	+++	++
Enriched fatty deposition (FD)	+	+++	+++	++
Congested blood sinusoids (BS)	-	+++	++	++
Dilated blood vessel (DBV)	-	+++	++	+
Necrotic areas (NA)	-	+++	+++	++
Enriched melano-macrophage centers (MMC)	+	++	+++	++

* Symbols indicate the total number of the lesion discovered in 9 examined fields; - no; + mild, ++ moderate, and +++ severe lesion.

II. Splenic pathology.

**Fig. 4:**
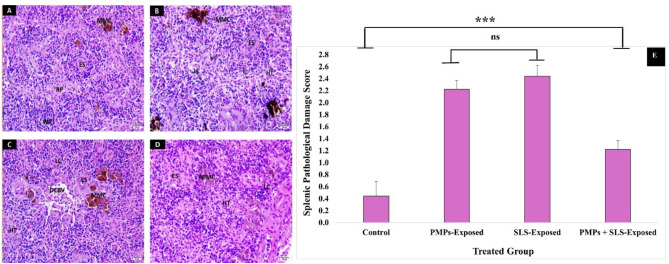
Photomicrographs of H & E-counterstained sections (×400; scale bar = 25 μm) of *C. gariepinus* spleen after 15 days of exposure to PMPs, SLS, or their combination. **(A)** A micrograph from a control fish demonstrates typical splenic tissue integrity with intact RP and WP. Limited MMC and ES patch were observed. **(B)** A micrograph from a fish subjected to PMPs demonstrates an increase in ES patch encircled by an RBC-containing sinus, MMC dispersed throughout the HT, and F. In addition, LC was noticeable. **(C)** A micrograph from a fish exposed to SLS displays severe deterioration in splenic tissue integrity. An increase in ES, HT, primarily RBC, and MMC, and a decrease in LC were obvious. In addition, DCBV were seen. **(D)** A micrograph from a fish subjected to PMPs + SLS shows minor histological assessments of MMC scattered throughout the HT around the ES, along with a depletion of LC and ES. **(E)** A bar graph displaying the severity score of the general splenic histopathological lesion ranging from 0 to 3. The data were estimated at 40x magnification and displayed as means ± SE of nine replicates for each group (*n* = 9). The statistically significant differences in values between groups (*P* < 0.05) are indicated by superscript symbols on the bars.

Figure 4, A displays the normal architecture of spleen tissue from the control group with white pulp (WP) consisting of aggregated lymphocytes and red pulp (RP) containing hemopoietic tissues mainly of RBC, MMC, and ellipsoid structures (ES), which lined by cubic cells. Spleen sections from the MP-exposed fish (10 mg/kg) (Fig. 4, B) show deformation of the splenic structure, and depletion of lymphocytes (LC). Increased patches of ES, which are surrounded by sinus-containing blood cells, and MMC were increased and distributed among the hemopoietic tissue (HT). Heavily drainage of hemopoietic tissues, mainly RBC, and acidophilic network fibers (F) were markedly increasing. The spleen sections from the SLS-exposed fish (4 mg/kg) (Fig. 4, C) showed dilated and congested blood vessels (DCBV) and small shape ES, depletion of LC, and increased HT, mainly RBCS. MMC were increased, distributed among the HT, and were observed surrounding the ES or near DBV. Spleen sections from the PMPs-and SLS-exposed fish (Fig. 4, D) show increased patches of ES surrounded by sinus-containing blood cells. MMC were increased and distributed among the HT and were observed surrounding the ES associated with depletion of LC, and increased HT, mainly RBCS. The scoring of the general pathological lesions observed in fish spleen is represented in a column histogram (Fig. 4, E). In addition, the differential scoring of the observed pathological lesions is collected in Table 5.

**Table 5:** The differential severity scoring of the observed spleen-pathological lesions after the exposure to PMPs and/or SLS.

Lesion	Group/score			
	Control	PMPs-treated	SLS-treated	PMPs + SLS-treated
Enriched ellipsoid structure (ES)	-	+++	+++	+
Encircled melano-macrophage centers (MMC)	+	+++	+++	++
Lymphocytic depletion (LC)	-	+++	+++	+++
Enriched hemopoietic tissues (HT) and RBCS	++	++	+++	+
Dilated and congested blood vessels (DCBV)	-	++	+++	+

* Symbols indicate the total number of the lesion discovered in 9 examined fields; - no; + mild, ++ moderate, and +++ severe lesion.

#### Histochemical analyses

I. Localization and quantification of fibrosis.

**Fig. 5:**
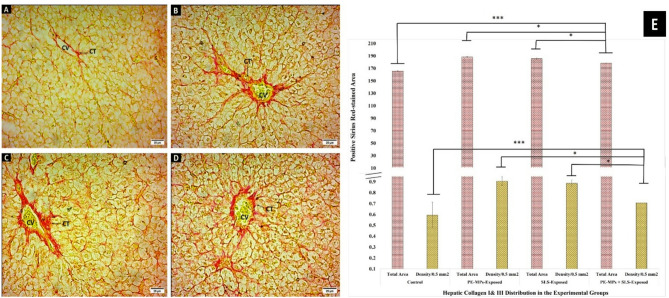
SR-staining findings of *C. gariepinus* liver after 15 days of exposure to PMPs, SLS, or their combination. **A-D)** Photomicrographs of SR-stained sections from *C. gariepinus* (×400; scale bars = 25 μm) showing: **(A)** A micrograph from the control group displaying the normal distribution of collagen types I & III colocalized around the CV with fine fibers between H. **(B)** A micrograph from a fish exposed to PMPs, and **(C)** a micrograph from a fish exposed to SLS show increasing levels of collagen depositions in the CT of the organ framework and around CV. **(D)** A micrograph from a fish exposed to PMPs + SLS demonstrates a decreased intensity of collagen I & III depositions in the CT surrounding the CV and between H in BS. **(E)** Column histogram displaying the density per 0.5 mm^2^ area and the expanding pattern (% area) of the hepatic collagen fibers (types I & III) in SR-stained liver sections from control, PMPs, SLS, or PMPs + SLS-exposed *C. gariepinus*. Using ImageJ software, the data were acquired and displayed as means ± SE of each group’s eighteen parts (*n* = 18). The statistically significant differences in values between groups (*P* < 0.05) are indicated by superscript symbols on the bars.

The liver sections of the control fish showed a normal distribution of collagen types I and III colocalized around the central vein with fine fibers between the H (Figs. 5, A). The intensity of collagen deposition was increased in fish exposed to PMPs (10 mg/L) and/or SLS (4 mg/L) (Figs. 5, B, and C). Compared to the other exposed groups, decreased intensity of collagen deposition was noticed in the fish exposed to the combined PMPs + SLS-exposed (Fig. 5, D).

Using ImageJ software, semi-quantifications of the microscopic observations of the hepatic fibrotic depositions from collagen fibers (types I and III) in the exposed groups were set up. Figure 5, E displays the statistical estimates of the density per 0.5 mm^2^ and distribution pattern (total area expansion) of hepatic collagen fibers. All exposed groups showed very high statistically significant levels of hepatic fibrosis (*P* < 0.001) when compared to the control group. Whereas the combination of PMPs + SLS-exposure revealed a significant reduction (*P* < 0.05) in fibrosis.

**Fig. 6:**
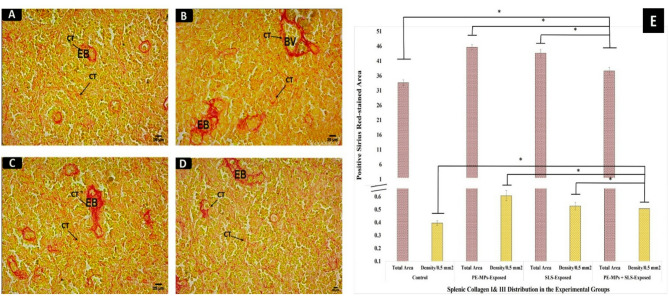
SR-staining findings of *C. gariepinus* spleen after 15 days of exposure to PMPs, SLS, or their combination. **A – D)** Photomicrographs of SR-stained sections from *C. gariepinus* (×400; scale bars = 25 μm) showing: **A)** A portion of a control spleen demonstrates the fine network of subterranean substances, blood vessels, and ES surrounded by scant CT fibers. **B**,** and C)** Sections of the spleen from fish subjected to PMPs or SLS demonstrate CT fiber accumulations around blood arteries, and ES. Fine collagenous networks are obvious in subterranean materials. **D)** A segment of a spleen from a fish exposed to PMPs + SLS demonstrates a visible decrease in the accumulation of collagen I and III fibers. **E)** Column histogram displaying the density per 0.5 mm^2^ area and the expanding pattern (% area) of the splenic collagen fibers (types I & III) in SR-stained spleen sections from control, PMPs, SLS, or PMPs + SLS-exposed *C. gariepinus*. Using ImageJ software, the data were acquired and displayed as means ± SE of each group’s eighteen parts (*n* = 18). The statistically significant differences in values between groups (*P* < 0.05) are indicated by superscript symbols on the bars.

Similarly, exposure to PMPs and/or SLS affects the spleen tissue integrity and shows signs of fibrosis. ES in the spleen tissue had a red tinge, as shown by the SR stain on collagen fibers in the CT of control fish (Fig. 6, A). Vascular walls and the splenic tissue matrix (BV) were shown to be covered in fine collagen fibers (arrow). Every exposed group showed increased fibrosis; most of the increases were seen in the groups exposed to SLS or PMPs (Figs. 6, B, and C). The group exposed to the combination of SLS and PMPs showed steadily decreased fibrosis (Fig. 6, D).

Using ImageJ software, the fibrotic depositions of collagen fibers (types I and III) in the exposed groups’ spleen were semi-quantified. Figure 6, E shows the statistically estimated total area expansion and density per 0.5 mm^2^ of the splenic collagen fibers. Like the liver, all exposed groups had significantly higher levels of splenic fibrosis (*P* < 0.05) compared to the control group, with the combined PMPs + SLS-exposed group showing a significant diminution in fibrosis (*P* < 0.05).

II. Localization and quantification of hepatic polysaccharide depositions.

**Fig. 7:**
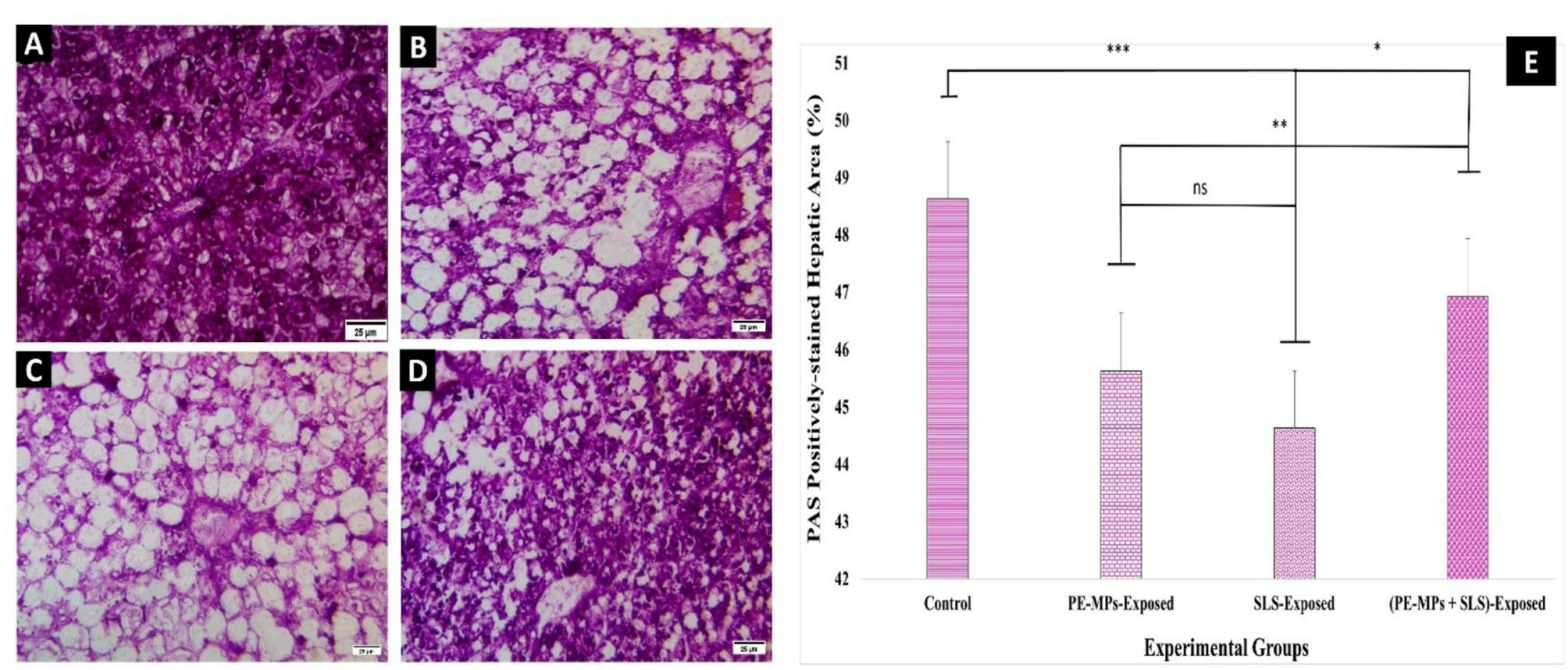
Photomicrographs (×400; scale bar 25 μm) of liver tissue from *C. gariepinus* stained by the PAS for polysaccharides showing: **A**) A micrograph from control fish showing the normal distribution of carbohydrates in H with the extensive glycogen. **B - C)** A micrographs from fish exposed to PMPs or SLS, showing glycogen depletion in H. **D)** A micrograph from combined PMPs + SLS-exposed group showing partially reversed glycogen content PAS reaction. **E)** A column histogram shows the growing pattern (% area) of the hepatic polysaccharides’ depositions in PAS-stained sections using ImageJ software. The data were acquired and displayed as means ± SE (*n* = 18). Superscript asterisks on bars denote the statistically significant different values of groups (*P* < 0.05).

Sections stained by PAS reagent displayed the polysaccharide localization in the liver (Fig. 7). In the control group, extensive reactivity of polysaccharides in H was observed (Fig. 7, A). A substantial reduction in the hepatocellular polysaccharide content was observed in PAS staining liver sections of the PMPs or SLS-exposed fish (Figs. 7, B, C). In liver sections from the combined PMPs + SLS-exposed group, partially reversed glycogen content was seen (Fig. 7, D).

In a bar histogram (Fig. 7, E), the semi-quantification of hepatic polysaccharide depositions in the experimental groups is statistically represented. The groups exposed to PMPs or SLS showed a significant decrease (*P* < 0.001) in the amount of glycogen deposits in the liver than the control group. Once more, the SLS-exposed group had the substantially lowest (*P* < 0.001) glycogen level whereas the PMPs + SLS-exposed group had the significantly highest (*P* < 0.05) glycogen level in comparison to the other groups. The glycogen depositions of the PMPs- and SLS-exposed groups did not differ statistically significantly (*P* > 0.05).

## Discussion

The current study was set out to evaluate the biological and immunological alterations in *C. gariepinus* exposed to PMPs and SLS, either singly or in combination. Physiological, immunological and haemato-biochemical characteristics were evaluated. In addition, tissue integrity biomarkers were investigated in liver and spleen.

Hematological indices are considerable indicators of the fish’s health condition^58^. According to Cole et al.^12^ MPs’ sharp surfaces can harm RBC membranes physically, resulting in cell lysis. This directly affects Hb levels and Hct values, especially with higher concentrations in blood, which accelerates hemolysis, disrupt erythropoiesis, destructs RBCs in the hematopoietic organ, or develops anemia^59^. Therefore, PMPs, SLS, and PMPs + SLS-exposed fish groups of our study showed a substantial decrease in RBC account, Hct, and Hb concentration. In agreement with our findings, a decrease in erythropoiesis and an increase in the rate of erythrocyte breakdown in hematopoietic organs, which could account for a notable reduction in RBCs, Hb, and HCT were reported in SLS-exposed catfish^60,61^. High level of MCV could be caused by an increase in the immature RBCs or by the presence of a significant number of older or larger RBCs^62^. According to the results of the current study, the MCH and MCHC levels in the PMPs, SLS, and PMPs + SLS-exposed groups are not significantly different compared to those of the control group. The MCHC level is an indicator of RBC shrinkage (increased MCHC) or swelling (decreased MCHC). Ultraviolet exposure induced marked RBC swelling and non-significant decreases in MCH^63^. The reduction in WBC count is suggestive of a weakened immune response and less stress after exposure to toxic substances. Therefore, the decrease in WBC count in our findings could be the consequence of MPs and SLS bioaccumulation inside tissues. According to Alkaladi et al.^64^, the decline in total WBC counts may be due to the deleterious impact of nanoparticles on the lymphoid tissues of the exposed fish. In addition, the physical blockage of the digestive tract by PMPs might lower nutrition absorption and hampers energy allocation has the potential to affect fish immune system^65^.

After 15 days of exposure to PMPs, SLS, or PMPs + SLS, the fish’s biochemical response increased the AST and ALT levels in serum. Many fish species have elevated AST and ALT in their circulation due to the hepatic potency of chemical contaminants, which typically results in cellular damage and necrosis in the hepatic tissues^19,66^. Alternatively, this elevation of ALT and AST activities can be an effort to supply the energy required to endure stress^67^. In our investigation, all exposed groups had low levels of ALP activity after 15 days of exposure to MPs, SLS, or both. Since ALP is an alkaline transphosphorylase that is necessary for biological membrane transport activities and skeleton mineralization, the reduced activity of the enzyme in fish serum caused by exposure to PMPs or SLS in the current study suggests disruptions in physiological and functional mechanisms linked to a defect in the membrane transport system^68^. In similar line to our findings, ALP activity was decreased in serum and other tissues of Nile tilapia in response to metal exposure in different ways^68–70^.

Here, the blood glucose levels significantly increased in the all the exposed groups of *C. gariepinus*, which indicates disturbances in glucose metabolism or uptake. This was further indicated by a reduction in the liver glycogen depositions. In agreement with our study, Hamed et al.^19^ reported a significant elevation of blood glucose of Nile tilapia *(Oreochromis niloticus*) exposed to MPs. In addition, the blood of exposed fish showed a considerable increase in the total protein level. This implies that the fish’s physical health, ability to function as an antioxidant, and immunological response were impacted by upsetting the overall protein influential balance. Furthermore, fish need to provide more energy in the blood to deal with stress, eliminate harm, and recover; therefore, serum protein levels rise and their liver proteins deteriorate^71^.

A significant rise in blood cholesterol levels was recorded in the current study. This may be because fish steroidogenesis utilizes cholesterol as primarily energy source since MPs and SLS may alter the circulation of fatty acids in the hepatic tissue, hence altering cholesterol levels. In a similar context, Hamed et al.^19^ reported that toxicants such as herbicides and MPs may alter the hepatic tissue’s fatty acid circulation, and perhaps affect cholesterol levels. Reversely, SOD and total antioxidant capacity activities in catfish serum showed a significant decrease indicating the negative reactivity for the fish’s antioxidant defense system following toxicant exposure, which increases the free radicals and reduces the overall antioxidant capacity. Comparable outcomes were documented utilizing antioxidant markers, which are extensively employed to assess the influence of distinct polyethylene plastic sizes (nano, micro, and macro) on juvenile C. *carpio* L^45^. Similarly, co-exposure of zebrafish to Cd and MPs revealed a reduction in SOD activity^72^. The reduce levels of antioxidant enzymes like SOD and CAT in blood indicates the oxidative stress attributed to increased lipid peroxidation in the liver^73^.

Increased activities of MDA were recorded in the current study, which indicate high lipid peroxidation. Oxidative stress and lipid peroxidation may be attributed to either malnutrition or inhibition of food digestion due to the ingested MPs by fish^72^. In addition, the release, absorption, or containment of potentially harmful compounds by MPs might also affect the immune system or the energy allocation and limit nutrient absorption^12^. In agreement to our finding, the marine Juvenile *Pomatoschistus microps* exhibited elevated levels of lipid peroxidation after being exposed to PMPs^74^.

Here, fish exposure to SLS and PMPs upset homeostasis and had pro-inflammatory effects, based on elevated blood cytokine levels. According to Wang et al.^75^, this is a common immune reaction to inflammation and cellular damage. Tissue injury impairs cellular homeostasis, and trigger interleukins as an instant immunological response to offset the increasing stressor^75^. In addition, the increased interleukin production is an adverse consequence of immunological defense and marked systemic inflammatory response^75,76^.

Notably, the morphology of erythrocytes was altered under a microscope, displaying a range of changes such as enlarged, sickle-shaped, teardrop-shaped, and visible vacuoles, as well as RBCs with a karyolysis signal. Fish erythrocyte changes have been noticed during hypoxic conditions^77^ and in response to agents such as radiation that cause death of the blood cells^78^. In addition, the existence of vacuoles in erythrocytes may be caused by an uneven distribution of hemoglobin, as noted by Ateeq et al.^79^. The observation of large blood cells was a common modification of their necrotic cellular boundaries. Because erythrocytes are essential for oxygen delivery, deformations of the erythrocytes can lead to low oxygen levels in the blood, which can aggravate or even induce respiratory disorders by affecting the circulatory system. Changes in the quantity and makeup of fish erythrocytes could result from this respiratory stress^80^. Additionally, pathological circumstances leading to the breakdown of RBCs may be displayed by fish exposed to toxins^81^^[,19^. concluded that the interaction of PMPs with erythrocytes limits the dehydrogenase of delta-aminolevulinic acid, which induces plasma membrane interruptions and produces poikilocytosis. Araújo et al.^82^ suggested that the increased generation of relative oxygen species (ROS) in erythrocytes may result from a direct interaction of their plasma membranes with the MPs.

Smaller-sized MPs are more likely to amass inside the fish body organs because they can transit past the digestive tract and into the blood circulation, but larger-sized MPs typically congregate in the digestive tract and are easily evacuated because of differences in size^83^. According to our investigation, toxic effects following exposure to PMPs, SLS, or both extend to the hepatic tissue integrity. As the primary organ involved in numerous metabolic processes, fish liver alterations in histology are now commonly employed as indicators of exposure to toxins and carcinogens. Hepatocytic injury may be associated with reduced energy and metabolic disruptions, disintegration of microtubules, inhibition of protein synthesis, and increased hepatic RBC formation^84^. Furthermore, physical blockage of hepatic capillaries by Polyethylene polymers translocated to the liver may cause sinusoidal dilatation and inflammatory cell infiltration, particularly when they are present in micro or nano sizes^85^. MPs that have accumulated and circulated inside fish tissues may have caused inflammatory responses, and the adherent immune cells would have produced more ROS as defense reactions to combat oxidation^86^. The oxidative stress revealed substantial reduction in SOD, CAT, GSH, and GST levels, which in turn caused kidneys and liver damage of toxified rats^73^. Our observation suggests that PMPs and SLS secreted pro-inflammatory chemokines and cytokines that boosted oxidative stress in fish’s immune-relevant organs and changed their immunological state by facilitating phagocytosis’s recognition and elimination of foreign antigens. The spleen experienced macrophage aggregation. As far as we know, the spleen is the only organ in the bony fish lymphoid organs that has a sizable number of macrophages and lymphocytes^87^. Here, histological analysis revealed large areas of fibrosis in the spleen’s trabeculae, indicating that the exposed fish had less parenchyma and more interstitial stroma. In addition, the tissues of fish exposed to these conditions displayed increased CT and pigmented macrophages associated with blood vessels. Previous studies have shown that fish exposed to heavy metals in various habitats also displayed significant levels of fibrosis, suggesting a relationship between toxicant exposure and the occurrence of fibrosis^88^^,89^.

Histochemical investigations revealed that the exposed groups showed a decrease in hepatic glycogen content, as indicated by PAS staining. This suggests either a decrease in the intestinal absorption of carbohydrates or an increase in the glycolytic activity required by a stressed-out, increased metabolism. This reduction in the liver glycogen might indicate disturbance of glucose metabolism, energy balance, or other glycogen-related processes. Moreover, SR-staining revealed that the groups exposed to PMPs or SLS had detrimental effects on the fibrotic texture of the splenic and hepatic tissues integrity. They revealed high levels of fibrosis either singly or in combination. However, the combined exposure had lower effect on liver glycogen and tissue fibrosis. Similarly, after the exposure to PMPs, a dose-dependently higher collagen depositions were displayed by Masson’s trichrome staining of fish liver^90,91^. In a similar line, Japanese medaka (*Oryzias latipes*) exposed to the toxicant “dimethyl nitrosamine” developed hepatic fibrosis and neoplasia^92^.

MPs and chemicals have a complicated relationship that probably depends on the chemical makeup, plastic quality, and the environmental factors. There are several pathways of the interaction between chemicals and MPs in water. In seawater, the sorption capacity of chemicals onto MPs is affected by the water ions, which enable the interaction of various surface groups on chemicals with MPs and modify their overall charge and/or characteristics^93^. The toxicity of the insecticide on rainbow trout (*Oncorhynchus mykiss*) was increased when it was sorbed onto polystyrene MPs^94^. Additionally, their muscle contents of protein, fatty acids, and amino acids were significantly altered^95^. Similarly, PMPs reduced the toxicity of chlorpyrifos insecticide on the microalga *Isochrysisg albana*^96^. However, in common carp (*Cyprinus carpio*) PMPs increased the toxicity of paraquat herbicide and changed the blood’s biochemical properties [98]. Hydrophobic partitioning is a key mechanism that has been the subject of numerous published studies. Chemical solubility plays a major role in the process of hydrophobic partitioning, which occurs when substances separate between an aqueous phase and a hydrophobic solvent, in this case, PMPs^97^. In the aquatic environments, chemical’s hydrophobicity hinders their dissolution, and therefore, they tend to be sorb onto other organic particles such as MPs^35^ Recently, SLS solution has been proven to interact with PMPs relative to their concentrations and medium acidity^98^.

## Conclusion

PMPs and SLS are hazards for fish altering their biology and pathology either singularly or in combination. Fish exposure to non-lethal doses of 10 mg/L PMPs and 4 mg/L SLS for 15 days deteriorated their biomarkers in blood and tissue. Unexpectedly, the severity of their harm was mitigated upon their combination, which indicate the antagonistic interaction between both reagents inside the biological systems. This was clear in the reduced hematologic and biochemical alterations, mild pathological appraisals, and the moderate fibrosis as well as hypoglycemia. Our findings indicate that excessive residues of plastics and detergents are considered a precursor for environmental hazards in aquaculture systems. Therefore, social processes, laws, and technological developments for the reduction of pollution caused by these reagents should be considered.

## Data Availability

The datasets used and/or analyzed during the current study available from the corresponding author on reasonable request.
